# Spontaneous Rupture of Splenic Hemangioma in a Neonate

**DOI:** 10.21699/jns.v5i3.365

**Published:** 2016-07-03

**Authors:** Bruno Martinez-Leo, Jorge Vidal-Medina, Jesús Cervantes-Ledezma, Arid Díaz De León-Rivera, Edith Díaz-Velasco

**Affiliations:** Division of Pediatric Surgery, Hospital Pediátrico Moctezuma, México City Health Secretariat and Faculty of Medicine, Universidad Nacional Autónoma de México

**Keywords:** Hemangioma, Benign splenic tumor, Circulatory shock, Rupture

## Abstract

Spleen vascular tumors such as hemangiomas, albeit rare, can present during neonatal period with unexplained circulatory shock. We present a case of a newborn with refractory hypovolemic shock and acute abdomen that underwent emergency splenectomy due to spontaneous rupture of a splenic hemangioma.

## CASE REPORT

A 24-day-old male baby presented to us with acute abdomen and circulatory shock. The baby was born vaginally to a healthy mother at term after an uneventful prenatal course. Several prenatal ultrasounds were performed and reported as normal. Apart from being small for gestational age (2,385 gr at 40 weeks of intrauterine age), birth and clinical histories were unremarkable and newborn and mother were discharged 24 hours after birth. Before presentation, the patient became suddenly pale, vomited once and was treated by his pediatrician with antiemetic drugs, lactobacilli and simethicone with slight clinical improvement. A few hours later, he again became irritable and taken to the referring hospital, where he was found to have signs of shock; he received red blood cell and fresh frozen plasma transfusions, respiratory support, and broad-spectrum antibiotics. Given the persistence of hemodynamic instability and tender abdomen, the patient was referred to our hospital suspecting bowel perforation. The patient was pale, with tender abdomen but there was no ecchymosis or palpable masses on abdominal examination. Ultrasound abdomen showed abundant free fluid. The caregivers categorically denied abdominal trauma or falls. Emergency laparotomy revealed hemoperitoneum and a laceration of the spleen at its lower pole (Fig. 1). Splenectomy was performed. Patient slowly recovered from shock and its clinical derangements, and was discharged uneventfully after 10 days. Penicillin was started and immunization against encapsulated agents was given at age 2 months. Histopathology analysis reported a ruptured cavernous hemangioma of the spleen (Fig. 2).

**Figure:**
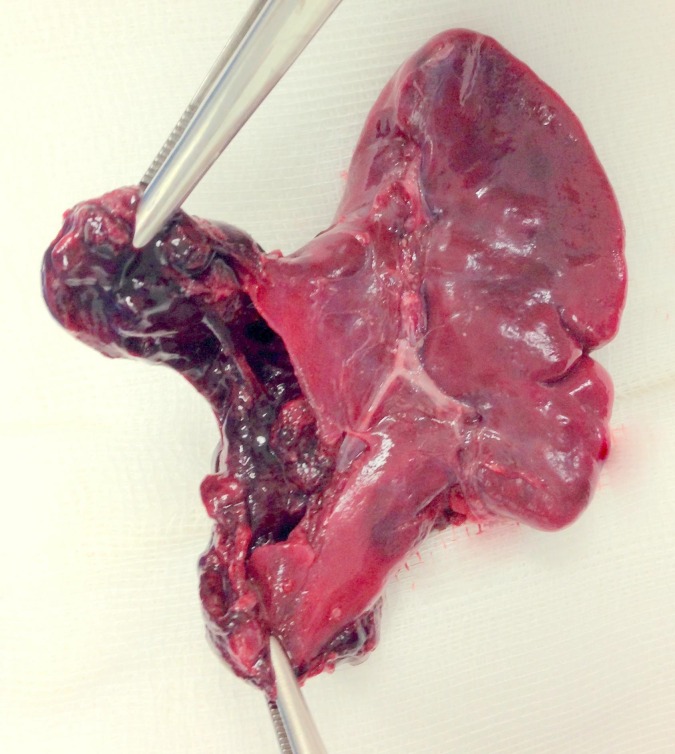
Figure 1: Resected spleen, showing a distorted anatomy and a ruptured lower pole.

**Figure:**
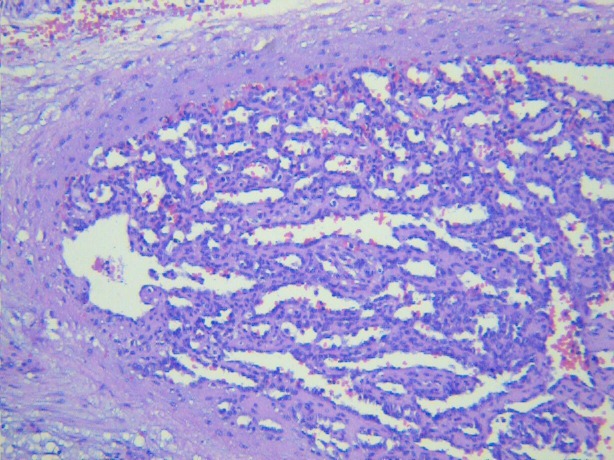
Figure 2: Hematoxylin and eosin slide showing cavernous hemangioma.

## DISCUSSION

The spleen can be involved by various conditions such as infectious processes, inflammatory diseases, vascular, or hematologic conditions and neoplasms (benign and malignant). The incidence of splenic hemangioma ranges between 0.02% and 0.16% [1,2]; it is seldom reported in the neonates. It may present as incidental lesions or it may produce significant splenomegaly and predispose to splenic rupture [3]. Other presentations include Kasabach-Merritt phenomenon, portal hypertension,[5] anemia, thrombocytopenia, coagulopathy and bleeding due to sequestration and/or destruction of blood components within the tumor [3]. Splenic hemangioma can have various complications such as splenic thrombosis, infarction, and spontaneous rupture which may be fatal if not dealt emergently [4]. 

Hypovolemic shock due to abdominal hemorrhage in neonates is very rare and can be caused by obstetric trauma, [6] laceration of umbilical artery during catheterization or unidentified causes [6,7]. Ruptured splenic hemangioma is extremely rare cause of abdominal hemorrhage [7,8]. Regardless of the cause of hemoperitoneum the morbidity and mortality can be very high if not identified and managed timely. Patients with splenectomy are prone to overwhelming infections, therefore, must be vaccinated as soon as possible, particularly against encapsulated microorganisms. In neonates, vaccination should be done at two months to get proper titers of antibodies [9]. 

To conclude, ruptured vascular malformation of spleen in neonates is very rare entity, and it must be considered when facing a neonate with unexplained shock and acute abdomen.

## Footnotes

**Source of Support:** Nil

**Conflict of Interest:** Nil

## References

[1] Burke JS. The Spleen. In: S.E. M, editor. Sternberg's Diagnostic Surgical Pathology. 4th ed. Philadelphia, PA: Lippincott Williams and Wilkins; 2004. p. 849-78.

[2] Willcox TM, Speer RW, Schlinkert RT, Sarr MG. Hemangioma of the spleen: presentation, diagnosis, and management. J Gastrointest Surg. 2000; 4:611-3. 10.1016/s1091-255x(00)80110-9 11307096

[3] Wilkins B, Wright DH. Illustrated pathology of the spleen. Cambridge, UK; New York, NY: Cambridge University Press; 2000.

[4] Abbott RM, Levy AD, Aguilera NS, Gorospe L, Thompson WM. From the archives of the AFIP: primary vascular neoplasms of the spleen: radiologic-pathologic correlation. Radiographics. 2004; 24:1137-63. 10.1148/rg.244045006 15256634

[5] Shanberge JN, Tanaka K, Gruhl MC. Chronic consumption coagulopathy due to hemangiomatous transformation of the spleen. Am J Clin Pathol. 1971; 56:723-9. 10.1093/ajcp/56.6.723 5166544

[6] Sokol DM, Tompkins D, Izant RJ, Jr. Rupture of the spleen and liver in the newborn: a report of the first survivor and a review of the literature. J Pediatr Surg. 1974; 9:227-9. 10.1016/s0022-3468(74)80127-2 4825796

[7] Rekha S, Lewin S, Lilly S, Vincent S, Ramachandra C, Chandrasekhara MK, et al. Hemoperitoneum secondary to splenic rupture in a neonate. Indian pediatr. 1992; 29:1575-6. 1291510

[8] Pachl M, Elmalik K, Cohen M, Kamupira S, Walker J, Murthi G. Ruptured splenic cavernous hemangioma in a neonate. J Pediatr Surg. 2008; 43:407-9. 10.1016/j.jpedsurg.2007.09.080 18280302

[9] Shiokawa S, Mortari F, Lima JO, Nunez C, Bertrand FE, 3rd, Kirkham PM, et al. IgM heavy chain complementarity-determining region 3 diversity is constrained by genetic and somatic mechanisms until two months after birth. J Immunol. 1999; 162:6060-70. 10229847

